# The Impact of Resonance Frequency Breathing on Measures of Heart Rate Variability, Blood Pressure, and Mood

**DOI:** 10.3389/fpubh.2017.00222

**Published:** 2017-08-25

**Authors:** Patrick R. Steffen, Tara Austin, Andrea DeBarros, Tracy Brown

**Affiliations:** ^1^Brigham Young University, Provo, UT, United States

**Keywords:** resonance frequency breathing, heart rate variability, blood pressure, mood, biofeedback

## Abstract

Heart rate variability biofeedback (HRVB) significantly improves heart rate variability (HRV). Breathing at resonance frequency (RF, approximately 6 breaths/min) constitutes a key part of HRVB training and is hypothesized to be a pathway through which biofeedback improves HRV. No studies to date, however, have experimentally examined whether RF breathing impacts measures of HRV. The present study addressed this question by comparing three groups: the RF group breathed at their determined RF for 15 min; the RF + 1 group breathed at 1 breath/min higher than their determined RF for 15 min; and the third group sat quietly for 15 min. After this 15-min period, all groups participated in the Paced Auditory Serial Addition Task (PASAT) for 8 min, and then sat quietly during a 10-min recovery period. HRV, blood pressure, and mood were measured throughout the experiment. Groups were not significantly different on any of the measures at baseline. After the breathing exercise, the RF group reported higher positive mood than the other two groups and a significantly higher LF/HF HRV ratio relative to the control group, a key goal in HRVB training (*p* < 0.05). Additionally, the RF group showed lower systolic blood pressure during the PASAT and during the recovery period relative to the control group, with the RF + 1 group not being significantly different from either group (*p* < 0.05). Overall, RF breathing appears to play an important role in the positive effect HRVB has on measures of HRV.

Heart rate variability (HRV) is a key marker of health, mood, and adaptation, and hence improvements in HRV improves health, mood, and adaptation to stress (1, 2). Heart rate variability biofeedback (HRVB) reliably increases HRV, mood, and adaptability (3, 4). A key aspect of HRVB involves identifying each person’s unique resonance frequency (RF) breathing rate and then teaching them how to breathe at this rate in the clinic and through home practice. No studies to date, however, have examined if breathing at RF is an important aspect of HRVB training. The purpose of this study was to examine whether different breathing rates would differentially affect measures of HRV, blood pressure, and mood. Specifically, does RF breathing result in more positive outcomes relative to other breathing rates?

Heart rate variability is the variation in time intervals between heart beats (5). Multiple physiological systems influence heart rhythm and greater fluctuations in heart rhythm over time indicate healthy systemic balance and ability to respond to physiological needs (3, 6). Consequently, higher levels of HRV are indicative of a healthy heart and a marker of overall healthy physiological functioning (5). Low HRV, or less responsiveness to physiological needs, predicts mortality and morbidity and also occurs in depression, anxiety, and chronic stress (7). As an example, the central nervous system uses negative feedback loops (such as the baroreflex) to maintain homeostatic balance in heart rate and blood pressure while interacting with the environment (1). However, these feedback loops become less sensitive with chronic stress and sympathetic nervous system arousal found in physical and psychiatric disorders (3). High heart rates are inversely correlated with the levels of HRV, as there is less opportunity for variability between heart beats when they are closer (5). Low HRV predicts negative outcomes in cardiovascular disease and in all-cause mortality (8), with depression playing a significant role in cardiovascular disease as well (1).

Heart rate variability biofeedback reliably improves HRV. Biofeedback is an interactive process where individuals directly learn how to change their physiological activity (9). Sensors placed on their skin measure physiological functions such as heart rate, respiration, muscle tension, etc., and this information is displayed on a computer screen in real time allowing participants to become directly aware of and then change their physiological functioning (5). HRVB is particularly beneficial in learning to regulate the physiological stress response. HRVB is a relatively quick and inexpensive treatment, with protocols typically including ten 30-min sessions, although positive effects on anxiety, depressive symptoms, and cognitive performance have been found after the first session (10–12). HRVB interventions are efficacious for both physical and mental disorders, including treating depression, anxiety, panic disorder, as well as improving outcomes in patients with cardiac problems and asthma (4,14,15).

A key aspect of HRVB is RF breathing. HRV is directly influenced by breathing and respiratory sinus arrhythmia (RSA). RSA is the fluctuation in heart rate corresponding to breathing, with heart rate increasing with inhalation and decreasing with exhalation (15). RSA typically occurs in the high frequency (HF) range of HRV (0.15–0.4 Hz) and is a measure of parasympathetic activity (16). At resting breathing rates (between 9 and 24 breaths/min), heart rate increases with inhalation at about the mid breath point and heart rate decreases with exhalation at about the mid breath point (5). RSA impacts gas exchange such that heart rate tends to be higher when the air in the lungs is richest in oxygen and exhalation occurs when carbon dioxide in the lungs is highest. Hayano et al. (17), however, found that the most efficient gas exchange occurs when heart rate starts increasing at the beginning of inhalation and decreasing as exhalation begins rather than at the mid breath points. Synchrony of heart rate and breathing also increases the amplitude of heart rate oscillations leading to high levels of HRV.

Heart rate and breathing synchronize, or become resonance, at about 6 breaths/min (0.1 Hz). Each person has a unique RF breathing rate, ranging typically between 4.5 and 7.0 breaths/min. In studies of HRV biofeedback, the most common RF breathing rate is 5.5 breaths/min (18). RF breathing rate is identified by having the person breathe at 4.5, 5.0, 5.5, 6.0, 6.5 and 7.0 breaths/min during EKG recording. HRV frequency and time domain measures are then evaluated to find which breathing rate results in the largest changes in HRV. A common approach is to examine the low frequency (LF) range (0.05–0.14) of the HRV spectrum to find the largest frequency spike, which usually occurs at about 0.1 Hz. As people slow their breathing down and approach RF, HRV amplitude increases significantly. When a person breathes at their identified RF breathing rate, heart rate and breathing become synchronized and the highest levels of HRV are typically obtained.

Although breathing at RF is a key part of HRVB training, there are no published studies to date that have examined if RF breathing is essential. We therefore tested the hypotheses that breathing at RF would lead to improved HRV, blood pressure, and mood using a randomized controlled design. Specifically, we examined three hypotheses. First, we hypothesized that the RF group would show higher positive mood and decreased negative mood and anxious arousal following breathing practice relative to the RF + 1 and control groups. Second, we hypothesized that the RF group would score higher on the HRV measures of LF and LF/HF ratio, and lower on HF relative to the RF + 1 and control groups. And third, we hypothesized that the RF group would show decreased blood pressure reactivity during the PASAT stressor relative to the RF + 1 and control groups, and that LF/HF ratio following breathing practice would predict decreased blood pressure reactivity during the PASAT stressor and that this effect would be strongest in the RF group.

## Method

### Participants

A convenience sample of 95 participants (60% female, average age of 20) was recruited from undergraduate psychology classes using an online recruitment site and randomized into three experimental groups: RF breathing, breathing at 1 breath/min above established RF, and a control group that sat quietly. Class credit was given for research participation. Participants were excluded if they reported a history of heart disease or taking medications that affect blood pressure or heart rate. This study was approved by a university institutional review board and all participants read and provided informed consent before starting the study.

### Measures

#### Heart Rate Variability

Heart rate variability was measured using both frequency domain measures (LF, HF, and LF/HF ratio) and time domain measures [standard deviation of normal to normal R-R intervals (SDNN) and Root Mean Square of the Successive Differences (RMSSD)]. HRV was measured using the Nexus 10 and Biotrace software (Mind media software). Data were corrected for artifact and heart variability measures were calculated using the Kubios program (University of Finland). Power spectral analyses (LF and HF) measures examine different frequencies: 0.15–0.4 Hz for HF, and 0.04–0.15 for LF (3, 19). The SDNN measures the standard deviation of the R spike to R spike. Larger SDNN values show more variation in heart rate (3, 19). RMSSD is a time domain measure of HRV, and measures the differences between adjacent heart rates. RMSSD correlated with vagus-mediated components of HRV (3, 19).

#### Blood Pressure

Data on heart rate, diastolic, and systolic blood pressure were collected using a Dinamp Model 8100 automated blood pressure monitor (Critikon Corporation, Tampa, FL, USA) that uses an oscillometric method. Readings were taken placing a cuff on the upper non-dominant arm of the participant following manufacturer specifications. Two blood pressure readings were averaged for each time period.

#### Mood and Anxious Arousal

The Scale of Positive and Negative Emotions (SPANE*)* was used to measure current mood. The SPANE consists of 12 items, 6 positive and 6 negative, to assess positive and negative emotions over a specific time frame. These are rated on a Likert scale from 1 (“very rarely or never”) to 5 (“very often or always”). The SPANE has demonstrated good internal consistency with Cronbach’s alphas between 0.81 and 0.89. It also correlates from 0.57 to 0.70 with other mood scales (20). The anxious arousal subscale was taken from the Mini Mood and Anxiety Questionnaire (21). The anxious arousal subscale contains 10 items and is designed to assess physiological symptoms associated with anxiety such as feeling short of breath, cold hands, or trembling muscles. Items are measured on a 5-point Likert scale, ranging from 0 (“Not at all”) to 5 (“Extremely”) and the subscale has been shown to have good internal consistency (α = 0.85).

### Procedure

After obtaining consent, participants were randomly assigned to the RF, RF + 1, or the control conditions. The experimental conditions consisted of breathing at RF, breathing at one breath above the RF (RF + 1), and a control group who are sitting quietly. Participants filled out an initial battery of measures, including the anxious arousal subscale from the Mini Mood and Anxiety Questionnaire, and SPANE. Researchers attached EKG sensors to participants’ wrists and forearm. The participant also wore a respiration belt and a blood pressure cuff on their non-dominant arm. RF was determined for all participants, used Rosenthal’s HRV determination protocol. The pacer was set at five different frequencies (7, 5, 6.5, 5.5, and 6 breaths/min) for 2 min, resting 1 min between each pace. Because previous studies failed to find students breathing at 4.5 breaths/min, this rate was not included in the present study. Participants then filled out self-report measures of typical stress responses. Participants in the biofeedback conditions then continued to breathe for 15 min, with those in the RF condition breathing at RF, and those in the RF + 1 group at one above their RF. Participants in the control condition sat quietly with their eyes open for 15 min. After this time, participants filled out self-report measures of stress and anxiety symptoms. All participants then took the Paced Auditory Serial Addition Task [PASAT (22)] as a brief laboratory stressor, followed by measures of mood and anxious arousal. Participants then sat quietly for 10 min, followed by measures of mood and anxious arousal, and were then thanked for their time.

### Data Analysis

The purpose of this study was to examine the impact of different breathing rates on physiological response using a randomized controlled design. We therefore used repeated measures analysis of variance to test the first two hypotheses, analyzing the impact of group membership on measures of heart variability, blood pressure, and mood during breathing exercises (or sitting quietly), during the PASAT stressor, and during a recovery period. We also used hierarchical linear regression to test the last hypothesis, examining if a measure of HRV (LF/HF) measured during breathing practice predicted blood pressure response to the PASAT stressor. All analyses were conducted using SPSS (IBM SPSS version 23). Three hypotheses were examined.

## Results

### Mood and Physiological Distress by Experimental Group

The experimental groups did not differ on gender composition or age, and there were no significant differences between groups on reported positive mood, negative mood, or physiological distress at baseline. The 3-Group × 4-Time ANOVAs on positive mood, negative mood, and physiological distress yielded significant main effects of time: *F*(3, 276) = 30.38, *p* < 0.001, η = 0.25 for positive mood; *F*(3, 276) = 30.55, *p* < 0.001, η = 0.25 for negative mood; and *F*(3, 276) = 16.19, *p* < 0.001, η = 0.15 for physiological distress, providing evidence that the experimental manipulations impacted the participants. Compared to baseline, positive mood fell significantly during the PASAT stressor and then recovered somewhat during the recovery period, whereas both negative mood and physiological distress increased from baseline to the PASAT stressor and then decreased during recovery.

The Group × Time interaction was significant for positive mood only, *F*(6, 276) = 3.44, *p* < 0.01, η = 07. Follow-up contrasts examining the changes from baseline to the end of the breathing practice showed that individuals in the RF group had increased positive mood whereas the other groups did not, *F*(2, 92) = 10.73, *p* < 0.001, η = 0.19. There were no significant Group × Time interactions for the PASAT and recovery periods.

### Measures of HRV by Experimental Group

The main hypothesis of this study was that different rates of breathing would differentially impact measure of HRV, with RF breathing leading to more beneficial results. The 3-Group × 5-Time ANOVAs on frequency domain measures of HRV (LF, HF, and LF/HF) yielded significant main effects of time: *F*(4, 372) = 15.78, *p* < 0.001, η = 0.15 for LF; *F*(4, 372) = 4.13, *p* < 0.01, η = 0.04 for HF; and *F*(4, 372) = 12.07, *p* < 0.001, η = 0.12 for the LF/HF ratio. The control showed little change over time in measures of HRV, whereas the RF and RF + 1 groups increased LF and decreased HF during breathing practice and decreased LF and increased HF during the PASAT stressor. Time domain measures of HRV (SDNN and RMSSD) did not show main effect differences over time nor were there Group × Time interactions.

**Figure 1:**
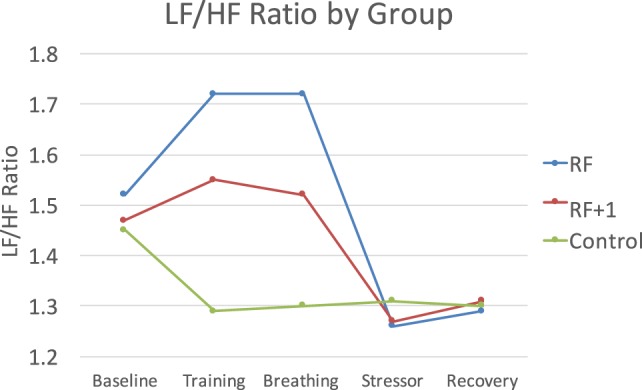
The RF group showed the largest LF/HF response to the breathing practice, with the RF + 1 showing minimal LF/HF changes and the control group decreasing during this time period.

The Group × Time interactions were significant for LF, HF, and the LF/HF ratio, *F*(8, 372) = 3.32, *p* < 0.001, η = 0.07 for LF, *F*(8, 372) = 2.18, *p* < 0.05, η = 0.05 for HF, and *F*(8, 372) = 3.96, *p* < 0.001, η = 0.08 for LF/HF. The RF group showed the largest changes during the breathing exercise, particularly for the LF/HF ratio which is a key variable tracked in HRVB. Follow-up contrasts examining changes from baseline to the end of the breathing practice revealed that the RF group increased in LF/HF ratio following breathing practice, whereas the RF + 1 group remained about the same and the control decreased, *F*(2, 93) = 4.64, p = 0.01, η = 0.09. Only the RF group was significantly higher than the control group, with the RF + 1 not being different from either group, indicating that a key therapeutic goal of HRV-B was most clearly achieved in the RF group (Figure 1).

### Impact of Experimental Group on Blood Pressure and Impact of LF/HF Ratio on SBP and DBP

We also hypothesized that RF breathing would result in lower blood pressure during breathing practice and reduced physiological responsiveness during a lab stressor. The 3 Group × 5-Time ANOVAs for SBP, DBP, HR revealed significant main effects with physiology decreasing during breathing practice and increasing the PASAT stressor, *F*(4, 292) = 36.71, *p* < 0.001, η = 0.34, for SBP; *F*(4, 292) = 13.86, *p* < 0.001, η = 0.16, for DBP; and *F*(4, 292) = 10.25, *p* < 0.001, η = 0.12, for HR, indicating that the experimental manipulations impacted physiology as expected.

**Figure 2:**
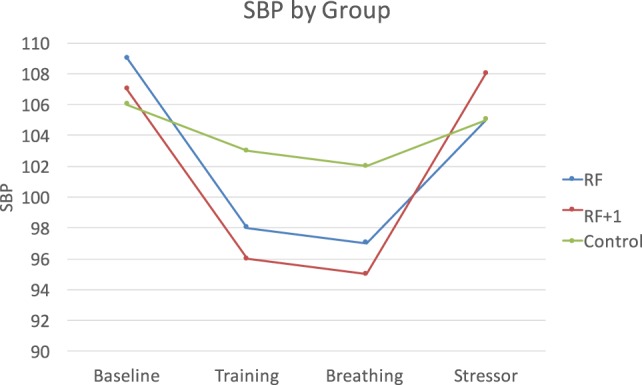
The RF and RF + 1 groups showed decreased blood pressure during breathing training compared to the control group. The RF group showed the smallest increase (least reactivity) in blood pressure in response to the stressor compared to the RF + 1 and control groups.

The Group × Time interactions were significant for SBP and DBP but not HR, *F*(8, 292) = 3.53, *p* < 0.001, η = 0.09, for SBP, and *F*(8, 292) = 3.69, *p* < 0.001, η = 0.09, for DBP. During breathing practice, both the RF and the RF + 1 groups showed lower SBP and DBP compared to the control group, *F*(2, 73) = 3.56, *p* < 0.05, η = 0.09 for SBP, and *F*(2, 73) = 4.94, *p* < 0.01, η = 0.12 for DBP. In response to the PASAT stressor, however, the RF group showed lower SBP compared to both the RF + 1 and control groups, *F*(2, 73) = 3.29, *p* < 0.05, η = 0.04 for SBP, suggesting that RF breathing buffered the stress response to the PASAT (Figure 2).

The final hypothesis was that LF/HF ratio would predict blood pressure response to the PASAT stressor. Using hierarchical linear regression, it was found that higher LF/HF at the end of breathing practice (controlling for baseline levels) predicted lower SBP during the PASAT (β = −0.21, *p* < 0.01) and during the recovery period (β = −0.28, *p* < 0.01). When examining effects by group, it was found that the effect was strongest in the RF group, β = −0.43, *p* < 0.02 for recovery period. Similar results were found for DBP with lower DBP found during recovery only (*r* = −0.26, *p* < 0.05), with the effect strongest for the RF group (*r* = −0.64, *p* < 0.05). These analyses indicate that the higher the LF/HF ratio, the less physiologically reactive people are to stress, with this effect being driven primarily by the RF group.

## Discussion

We examined whether breathing at RF would improve HRV, blood pressure, and mood, compared to breathing 1 breath/min above RF and sitting quietly using a randomized controlled design. Following the breathing exercise, the RF group reported the highest positive mood and showed a higher LF/HF ratio. Whereas the RF group increased significantly in LF/HF ratio, the RF + 1 group did not change significantly and the control group decreased. The LF/HF ratio is a key variable in HRVB and only the RF group displayed the increase that is sought for. The RF group also showed larger reductions in BP, particularly in response to the PASAT stressor. We also examined whether LF/HF ratio at the end of breathing practice would predict less BP reactivity during the subsequent PASAT stressor. Higher LF/HF did predict lower BP reactivity and this effect was seen most strongly in the RF group. Overall, results indicate that RF breathing contributes to healthier physiological response and mood, supporting its use in HRVB specifically and stress reduction generally.

Resonance frequency breathing is a key part of HRVB protocols (3). A number of research studies, however, have found positive results only emphasizing slow breathing or breathing at 6 breaths/min (23–25). Lin et al. (23) had healthy college students breathe at 6.0 and 5.5 breaths/min and found that the 5.5 rate resulted in higher HRV. RF breathing rate was not determined, rather everyone breathed at the same rate. Mason et al. (24) found that breathing at 6 breaths/min as part of yoga exercise improved oxygen saturation and baroreflex sensitivity. Zautra et al. (25) examined fibromyalgia pain patients when they were breathing normally and when breathing at half their normal rate and found slower breathing was related to decreased pain and depressive symptoms. A key finding of this study is that breathing at RF does matter. It is not clear if this would be the case in all situations, but there is evidence that HRVB and RF breathing results in stronger effects (4). Future studies could continue this line of research by examining slow breathing versus RF breathing with different populations and diagnoses to examine these possibilities.

Vaschillo et al. (18, 26, 27) documented that in addition to being an RF for breathing, there is also an RF for vascular tone, and that these resonance frequencies interact. Therefore, vascular tone may be impacted by RF breathing in terms of BP response to stress, with RF breathing decreasing BP and reducing BP response during stress. That is what was found in this study. The RF group increased their LF and HF/LF ratio following breathing practice, and this predicted decreased BP during the PASAT stressor. This confirms earlier research that those with higher HRV are more physically and emotionally resilient (5), but goes one step further to show that this can occur after one 15-min session RF breathing session.

Heart rate variability biofeedback potentially creates favorable outcomes in physical and mental health disorders through several mechanisms of action. Recent research has explored how strengthening and activating the baroreflex and vagal nerve increase physical and emotional resilience (3). Increases in LF and the HF/LF ratio indicate that these systems are being activated and strengthened. Breathing at RF, versus breathing near or slightly above RF, produced the highest LF/HF ratio, which can be interpreted as higher levels of baroreflex and vagal nerve activity. As these are proposed mechanisms of action for the reduction of symptoms in physical and psychological disorders, breathing at RF will produce greater effects in HRV interventions.

A limitation of this study is the cross-sectional design. While positive changes were seen with only one session, it is unknown what would happen over the course of weeks as HRVB is typically administered. Additionally, the sample consisted of college students, so it is unknown if these results would be found in different age groups. We did not assess baroreflex in this study which would have been helpful in understanding the pathways through which HRV changes occurred. A strength of this study was the use of a randomized controlled design with participants not being significantly different on the variables assessed at baseline. Therefore, there is confidence that the changes observed after exposure to the experimental conditions was not due to random effects.

## Conclusion and Future Directions

Overall, evidence supports RF breathing as a key factor in HRVB. RF breathing, compared to breathing at 1 breath above RF and sitting quietly control group, leads to more positive outcomes, resulted in a more positive mood, a higher LF/HF ratio (a key variable in HRVB), and a decreased BP response to stress. This study contributes to research on HRV showing that breathing at RF, as opposed to breathing near RF, promotes more adaptive physiological and emotional response. Future directions in this line of research include examining these relationships longitudinally over the course of HRVB, to study older age groups, and to explore if these relationships hold in people with clinical diagnoses such as depression or anxiety.

## Ethics Statement

This study was carried out in accordance with the recommendations of Brigham Young University Institutional Review Board with written informed consent from all subjects. All subjects gave written informed consent in accordance with the Declaration of Helsinki. The protocol was approved by the BYU Institutional Review Board.

## Author Notes

Patrick R. Steffen, Ph.D., Department of Psychology, Brigham Young University.

## Author Contributions

PS was involved with study conceptualization, design, statistical analysis, and writing up the manuscript. TA was involved with study design, analysis, data collection, and writing. AD and TB were involved with design, analysis, and data collection.

## Conflict of Interest Statement

The authors declare that the research was conducted in the absence of any commercial or financial relationships that could be construed as a potential conflict of interest.
